# Enrichment and characterization of steroid-degrading microbes for targeted removal of steroid hormone micropollutants in small-scale wastewater treatment solutions

**DOI:** 10.1128/spectrum.00649-25

**Published:** 2025-09-25

**Authors:** Katharina Kujala, Laura Lucia Nübel, Bodo Philipp, Johannes Holert

**Affiliations:** 1 Natural Resources Institute Finland 419837, Oulu, Finland; 2 Water, Energy and Environmental Engineering Research Unit, University of Oulu 6370, Oulu, Finland; 3 Institute of Molecular Microbiology and Biotechnology, Microbial Biotechnology & Ecology research group, University of Münster 9185, Münster, Germany; 4 Environmental Microbiology, Fraunhofer Institute for Molecular Biology and Applied Ecology 683029 , Schmallenberg, Germany; Commonwealth Scientific and Industrial Research Organisation, Brisbane, Australia

**Keywords:** bioaugmentation, steroid hormones, onsite wastewater treatment, *Comamonas*, micropollutants

## Abstract

In remote areas, wastewater treatment typically occurs directly at the source in decentralized systems, which often work well in removing biochemical oxygen demand, nitrogen, and phosphorus, but not micropollutants like pharmaceuticals and steroid hormones. Microorganisms capable of degrading such micropollutants could be used in bioengineered systems to increase micropollutant removal. In this study, bacteria were enriched from municipal wastewater in flow-through reactors supplemented with testosterone (TE), androsta-1,4-dien-3,17-dion (ADD), 17β-estradiol (E2), or 17α-ethinylestradiol (EE2). The removal of TE and ADD was stable over multiple transfers, and several androgen-degrading *Comamonas* strains were isolated. In contrast, the removal of E2 and EE2 was considerably slower, and no estrogen-degrading strains were isolated, suggesting that estrogens were removed mainly by adsorption and precipitation rather than by biological activity. The system’s complexity was then increased to approach real-life conditions by increasing reactor size, adding lactate and acetate as alternative carbon sources, and/or sterilized real wastewater and supplementing a hormone mix. Sequencing-based community analysis confirmed *Comamonas* as the main androgen degrader in these reactors but did not identify any known estrogen degraders. Alternative carbon, but not real wastewater, reduced hormone removal rates. These results show that (i) efficient androgen-degrading bacteria can be readily isolated using flow-through reactors and low hormone concentrations, while isolation of estrogen degraders is more difficult, (ii) androgen-degrading *Comamonas* strains are good candidates for bioaugmentation of small-scale water treatment solutions, and (iii) alternative carbon sources might hinder steroid degradation. This provides a basis to improve steroid removal from wastewater by bioaugmentation to prevent hormone discharge into water bodies.

**IMPORTANCE:**

Steroid hormones are endocrine-disrupting compounds exhibiting adverse effects on humans and the environment even at very low concentrations. Their removal during water treatment is often insufficient, and novel methods are required to increase hormone micropollutant removal from wastewater. This study enriched and isolated steroid hormone-degrading microorganisms in a flow-through system and tested their usability for bioaugmentation of small-scale water treatment solutions. While removal of estrogen hormones was found to be mainly abiotic, efficient biological androgen degradation was achieved. The combined results show that flow-through reactors, along with appropriate surface materials, are suitable for isolating biofilm-forming and hormone-degrading bacteria and that these microbes can be used for bioaugmentation to remove low concentrations of steroid hormones from wastewater. Based on these results, recommendations for future enrichment studies for bioaugmentation are formulated, and potential pitfalls are discussed.

## INTRODUCTION

In the last decades, the occurrence of organic micropollutants in natural environments, including personal care products, steroid hormones, industrial chemicals, and pesticides, has emerged as an important issue (1, 2). Many micropollutants are considered “contaminants of emerging concern,” as their environmental impacts are not yet well understood and challenging to foresee due to a lack of data regarding their occurrence, fate, and toxicity needed for a comprehensive assessment (3, 4). The main concern is that imperceptible effects can gradually accumulate, finally leading to irreversible changes in both wildlife and humans (5).

The point of entry of the majority of micropollutants into surface water sources (rivers, lakes, etc.) is sewage discharge. In sparsely populated areas, where the establishment of conventional wastewater treatment plants (WWTPs) is not feasible, small-scale on-site sewage treatment facilities are frequently used. Removal of organic pollutants in large- and small-scale treatment systems occurs mainly via biological and/or chemical transformation and sorption (3). However, removal of micropollutants is often incomplete, as many of these chemicals are recalcitrant and/or toxic to microorganisms (6). Even some organic molecules for which effective microbial degradation pathways exist, such as steroid hormones (7, 8), are often not fully degraded during wastewater treatment and remain in the discharge as micropollutants. Moreover, chemical processes and biological degradation of micropollutants can lead to the formation of transformation products, which, in turn, can be harmful in themselves. Thus, complete degradation of micropollutants to CO_2_ and biomass or their transformation into harmless products is preferable for the safe removal of those compounds.

To enhance the biodegradation of micropollutants, bioaugmentation of water treatment facilities is increasingly being considered, which implies the establishment of microorganisms in the water purification processes that have been specifically isolated for their ability to efficiently degrade or transform micropollutants into harmless end products (6, 9, 10). The addition of bioaugmentation strains or mixed cultures to bioengineered systems is expected to improve the degradation of the target compounds and improve their removal (11–13). Bioaugmentation of wastewater treatment systems has been suggested as a feasible technology for enhancing the removal of organic micropollutants, nitrogen, or phosphorus in natural and conventional systems (6). However, only a limited number of field applications of bioaugmentation have been reported to date (14, 15). Development of technology and methodologies in this field can lead to cost-effective, less energy-intensive, and sustainable solutions for micropollutant removal compared to methods applied in conventional WWTPs.

**Fig 1:**
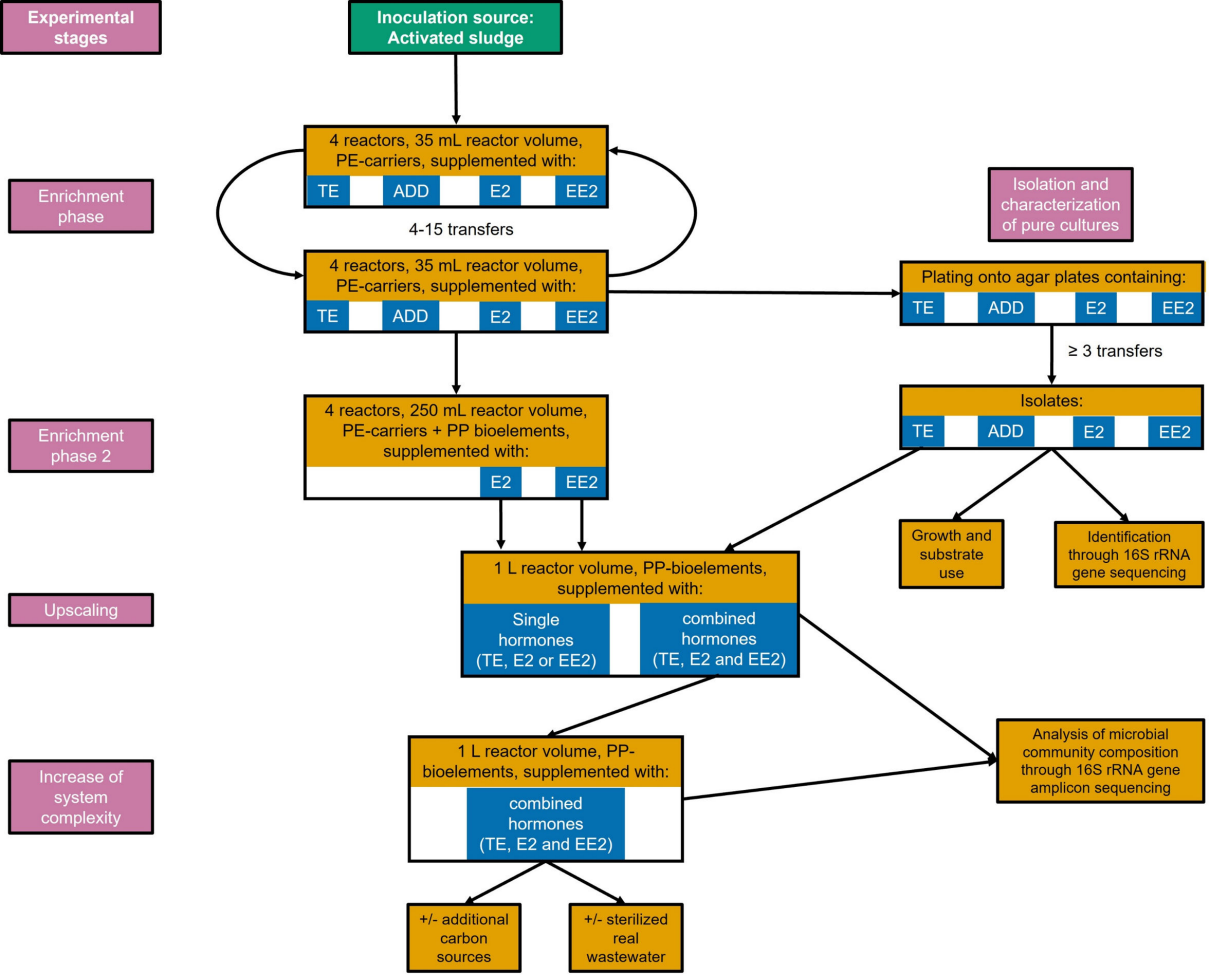
Overview of the experiments conducted in the present study. TE = testosterone, ADD = androsta-1,4-dien-3,17-dion, E2 = estradiol, EE2 = ethinylestradiol. For upscaling to 1 L, bioreactors with TE were inoculated with testosterone-degrading isolates, while bioreactors with E2 and EE2 were inoculated with PP-bioelements from enrichment phase 2.

The current study investigated the possibilities of utilizing highly efficient androgen and estrogen hormone degraders in wastewater treatment systems, aiming to test bioaugmentation with microorganisms capable of micropollutant removal on the laboratory scale. The study aimed to (i) specifically enrich biofilm-forming and steroid hormone-degrading strains or consortia that can be used for bioaugmentation of existing wastewater treatment solutions, (ii) test the potential of these strains or consortia to degrade steroid hormones in flow-through bioreactors under conditions that are commonly found in wastewater treatment systems, and (iii) characterize the microbial communities in the bioreactors. To accomplish these aims, the study conducted a series of enrichment steps in artificial wastewater (AWW) medium in small-scale reactors with only one hormone supplemented in each reactor. The systems’ complexity was then increased stepwise by supplementing a combination of hormones and by adding non-steroid carbon sources or sterilized activated sludge (AS; Fig. 1).

## RESULTS

### Enrichment of steroid hormone-degrading microorganisms

**Fig 2:**
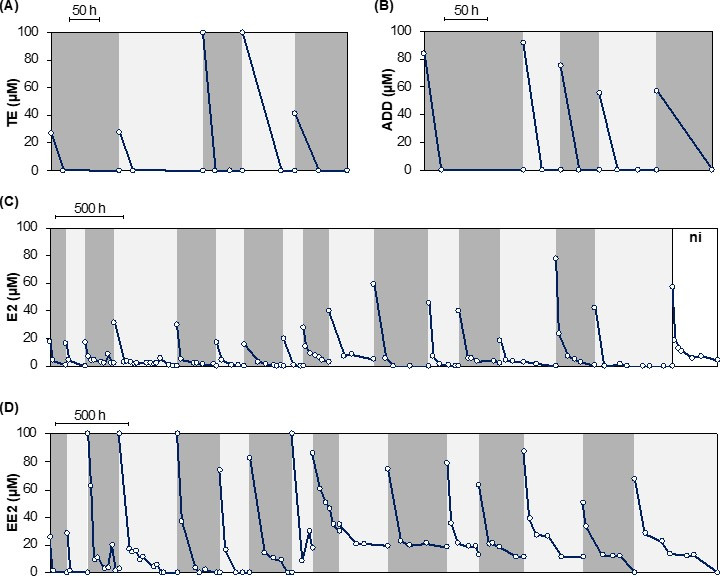
Removal of (**A**) TE, (**B**) ADD, (**C**) E2, and (**D**) EE2 in flow-through reactors for up to 4 transfers for androgen substrates and up to 15 transfers for estrogen substrates. Consecutive transfers are marked alternately in a gray background color. For the E2 enrichment, an uninoculated control (ni) was set up in parallel with the 14th transfer and is marked with a white background.

To enrich microorganisms capable of removing low concentrations of steroid hormones from wastewater, multi-step enrichment cultures were successfully established in small 35 mL flow-through bioreactors containing the androgen hormones testosterone (TE) or androsta-1,4-dien-3,17-dion (ADD), or the estrogen hormones 17β-estradiol (E2) or 17α-ethinylestradiol (EE2) as growth substrates and polyethylene (PE) carrier plates for biofilm formation (Fig. 1). For the selection of biofilm-forming and steroid hormone-degrading microorganisms, biofilms grown in androgen enrichments were transferred to fresh medium four times in total, while biofilms grown in estrogen enrichments were transferred 15 times. Complete or near-complete removal of all four hormones was observed throughout all enrichment steps (Fig. 2), except in some transfer steps of the EE2 enrichment, in which around 10%–30% of EE2 remained in the culture supernatants. While TE and ADD were depleted after 20–65 hours of incubation (Fig. 2A and B), E2 and EE2 were removed more slowly and were typically depleted after 50–250 hours (Fig. 2C and D). In E2 and in some EE2 enrichments, only around 15%–80% of the added substrate was detected right after inoculation of each enrichment culture, suggesting that abiotic effects were responsible for this initial drop in E2 and EE2 concentration. However, a steady removal activity of the remaining E2 and EE2 was observed in all enrichment steps, and E2 was completely depleted in most enrichments. In addition, final E2 levels in most enrichment cultures were lower than in an uninoculated control (Fig. 2C), suggesting additional biological E2 removal activity in the enrichments. No UV-absorbing transformation products or degradation intermediates were detected in any culture over the incubation time.

In the androgen enrichments, strong biofilm formation was observed on PE carrier plates throughout the four enrichment steps. Planktonic growth decreased and biofilm formation increased with increasing transfer steps in these cultures (Fig. S1A and B). In E2 and EE2 enrichments, biofilm formation on PE carrier plates was observed, but to a lesser degree than for TE and ADD, while planktonic growth increased with increasing transfer steps (Fig. S1C and D). A control culture without hormone, which was inoculated from the fourteenth E2 enrichment step, showed very strong planktonic growth (Fig. S1C), suggesting microbial growth with other carbon sources in the AWW medium. The lower prevalence of biofilm-forming microorganisms might have contributed to the observed lowering in EE2 removal in the later transfers, as only plastic carriers were transferred.

### Isolation of hormone-degrading strains

After three enrichment transfers, hormone-degrading bacteria were isolated from all four enrichments from biofilm-containing PE carrier plates. For each hormone substrate, several isolates were taxonomically classified based on 16S rRNA gene sequencing and were tested for steroid hormone degradation in liquid cultures.

**Fig 3:**
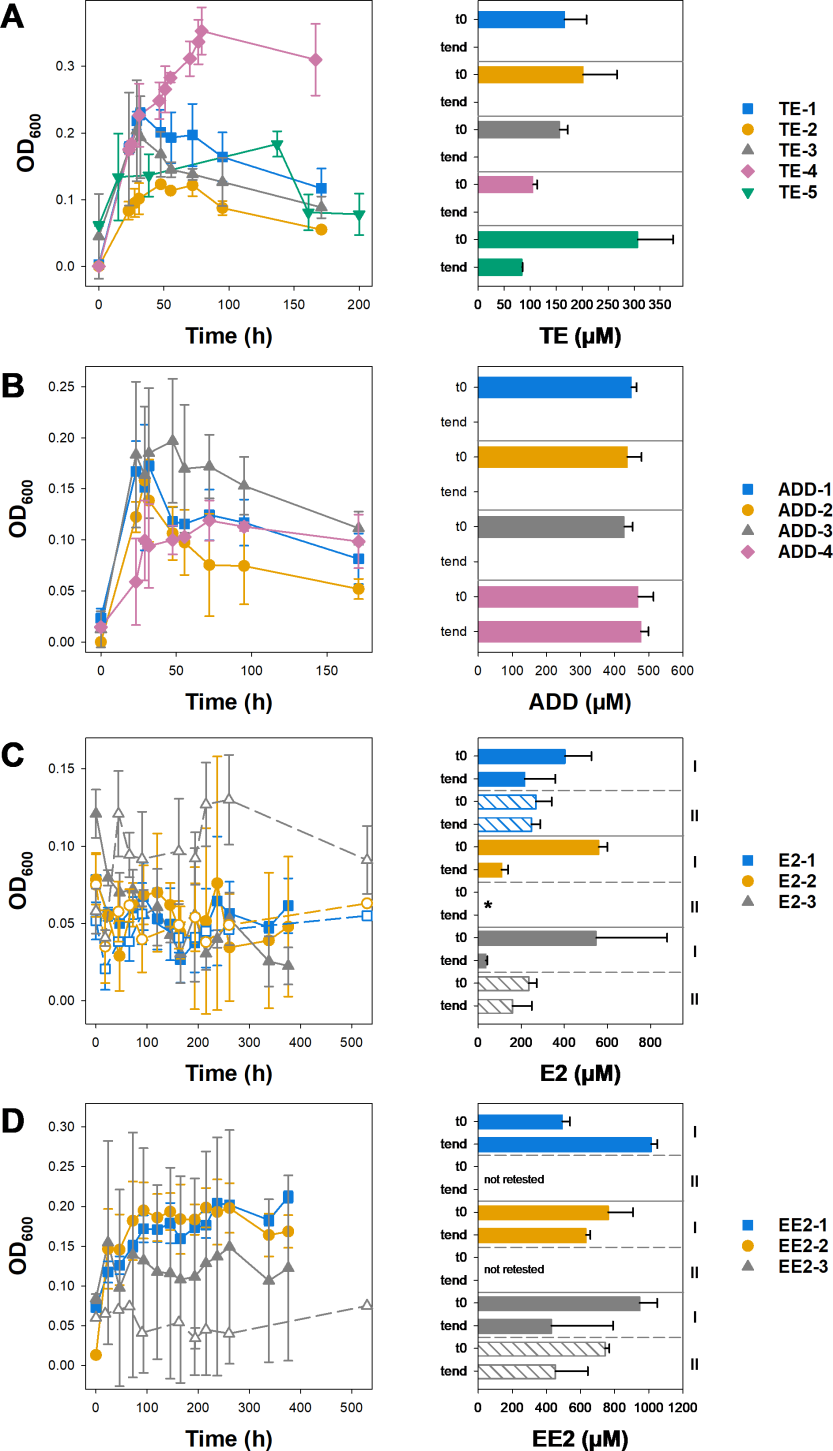
Growth and substrate removal of isolates from enrichment cultures with their respective isolation substrate in 5 mL cultures in glass tubes. (**A**) TE, (**B**) ADD, (**C**) E2, and (**D**) EE2. Growth was followed by measuring OD_600_. Substrate concentrations were determined at the beginning and the end of the growth experiments. Growth curves (solid symbols) and substrate consumption (solid bars) were tested in duplicate for all isolates. Selected E2 and EE2 isolates were retested in triplicate (open symbols and bars). Average and standard deviation of two or three replicates are displayed. *, substrate use was not determined, as no growth was observed for this isolate in the second test.

Originally, four TE isolates were obtained, three of which were classified as *Comamonas* sp. strains (TE-1–TE-3; Table 1; Fig. S2) and the fourth as a *Zoogloea* sp. strain. While the latter isolate originally showed good TE degradation characteristics in liquid culture (not shown), it was later shown to be a mixed consortium consisting of another *Comamonas* sp. strain (TE-4; Table 1; Fig. S2) and a *Pseudomonas* sp. strain (TE-5; Table 1; Fig. S2), which were again tested for their ability to metabolize TE. All four *Comamonas* isolates grew with and degraded 500 µM TE in liquid culture (Fig. 3A). In contrast, although *Pseudomonas* sp. strain TE-5 showed growth in AWW medium containing TE, TE was not removed completely from the cultures (Fig. 3A). Three of the four strains isolated on ADD were identified as *Comamonas* sp. strains and one as a *Pseudomonas* sp. strain (Table 1; Fig. S2). Again, all *Comamonas* sp. strains (ADD-1–ADD-3) showed growth in AWW medium with ADD and completely removed ADD from the growth medium within seven days, while *Pseudomonas* sp. strain ADD-4 showed growth in AWW but did not remove the ADD substrate (Fig. 3B).

**TABLE 1:** Taxonomic classification of isolates from TE, ADD, E2, and EE2 enrichment cultures and their capability to degrade their respective isolation substrate.

Isolation substrate	Isolate ID	LCA (SILVA)	Closest relative (NCBI 16S ribosomal RNA database)			Hormone substrate degradation
			ID	Accession	Identity (%)	
TE	TE-1	g_Comamonas	*Comamonas testosteroni* KS 0043	NR_029161.2	99.86	Yes
	TE-2	g_Comamonas	*Comamonas testosteroni* NBRC 14951	NR_113709.1	99.86	Yes
	TE-3	g_Comamonas	*Comamonas testosteroni* KS 0043	NR_029161.2	100.00	Yes
	TE-4	g_Comamonas	*Comamonas testosteroni* KS 0043	NR_029161.2	100.00	Yes
	TE-5	g_Pseudomonas	*Pseudomonas urethralis* BML-PP042	NR_181197.1	99.90	No
ADD	ADD-1	g_Comamonas	*Comamonas testosteroni* KS 0043	NR_029161.2	100.00	Yes
	ADD-2	g_Comamonas	*Comamonas testosteroni* KS 0043	NR_029161.2	100.00	Yes
	ADD-3	g_Comamonas	*Comamonas testosteroni* NBRC 14951	NR_113709.1	100.00	Yes
	ADD-4	g_Pseudomonas	*Pseudomonas sichuanensis* WCHPs060039	NR_180102.1	100.00	No
E2	E2-1	g_Zoogloea	*Zoogloea resiniphila* DhA-35	NR_027188.1	98.23	No
	E2-2	f_Comamonadaceae	*Pelomonas saccharophila* NBRC 103037	NR_114189.1	99.12	No
	E2-3	g_Pseudomonas	*Pseudomonas urethralis* BML-PP042	NR_181197.1	99.56	No
EE2	EE2-1	Unclassified	*Comamonas testosteroni* KS 0043	NR_029161.2	99.34	No
	EE2-2	g_Methyloversatilis	*Methyloversatilis universalis* FAM5	NR_043813.1	99.10	No
	EE2-3	g_Comamonas	*Comamonas testosteroni* NBRC 14951	NR_113709.1	99.64	No

Strains isolated with E2 and EE2 were affiliated with the genera *Pseudomonas, Zoogloea*, *Pelomonas*, *Methyloversatilis,* and *Comamonas* (Table 1; Fig. S2). While none of the E2 isolates showed an increase in OD_600_, although E2 concentrations decreased in some cultures (Fig. 3C), most EE2 isolates showed growth without a concomitant decrease in the EE2 concentrations (Fig. 3D). In summary, none of the estrogen isolates showed reproducible removal of its respective isolation hormone or reproducible growth characteristics, suggesting that these isolates were not capable of steroid degradation in pure culture.

### Upscaling of hormone-degrading enrichments to 1 L flow-through bioreactors

**Fig 4:**
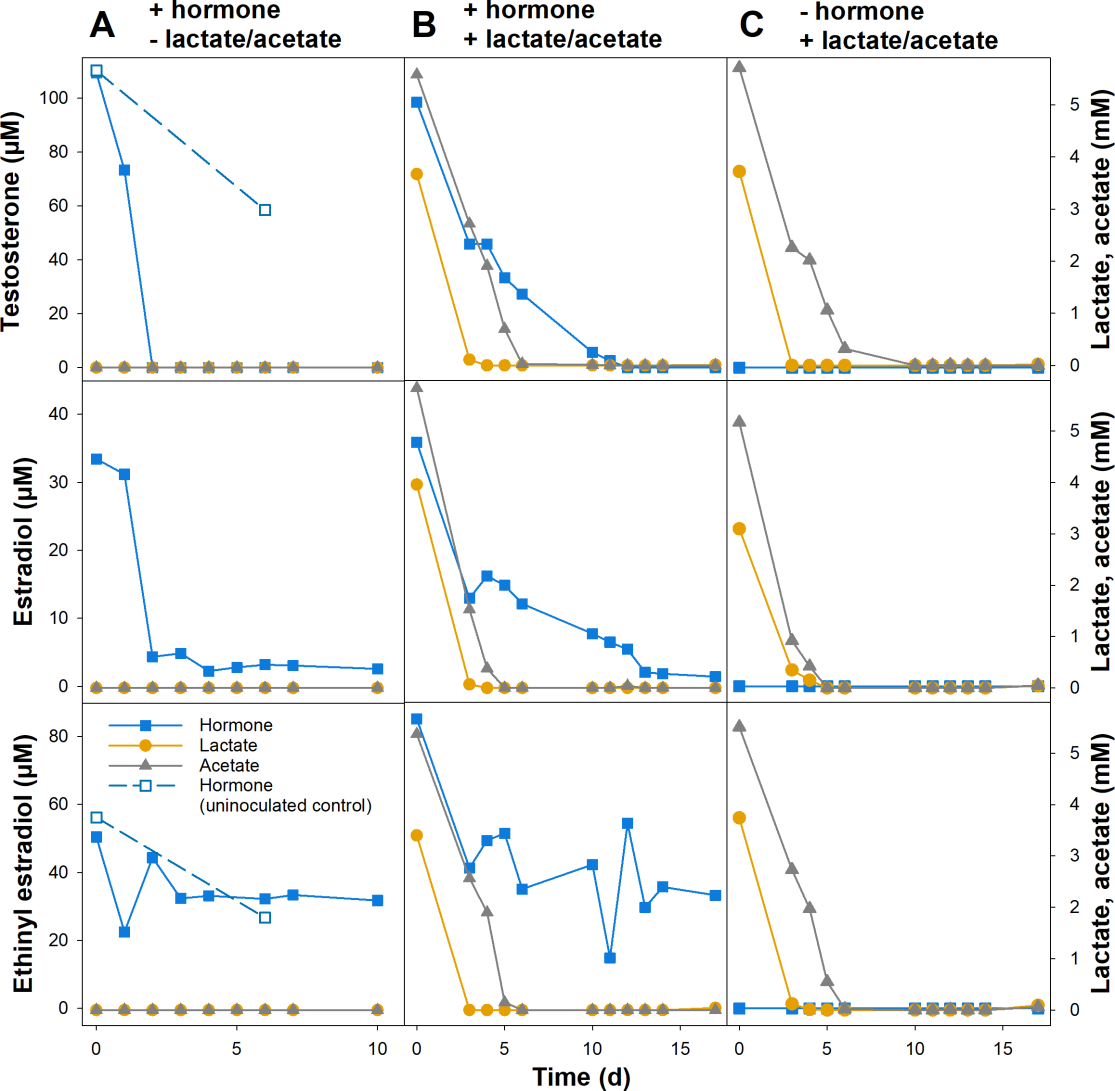
Degradation of TE, E2, and EE2 in upscaled flow-through bioreactors. Reactors were supplemented with 100 µM hormone only (**A**), with 100 µM hormone and 5 mM lactate and acetate (**B**), and with 5 mM lactate and acetate only (**C**). Hormone concentrations in uninoculated controls (**A**) were determined only twice during the incubation.

In the next step, bioreactors were upscaled to 1 L systems with TE, E2, and EE2. The TE-containing bioreactor was inoculated with colonies of the *Comamonas* sp. strain TE-4 and *Pseudomonas* sp. strain TE-5 consortium isolated from TE enrichments. Since none of the isolated strains from the E2 and EE2 enrichments showed consistent hormone degradation in pure culture, the E2- and EE2-containing bioreactors were inoculated with five biofilm-containing bioelements from intermediary medium-scale 250 mL enrichment bioreactors containing the same hormone (Fig. 1). The latter were inoculated with biofilm-containing bioelements from the respective eighth small-scale enrichments transfers. In bioreactors with single hormones, TE and E2 were removed within 2–3 days, and TE removal was significantly faster and more pronounced than in an uninoculated control (Table 2; Fig. 4A). A UV-absorbing TE transformation product with an absorption maximum at 245 nm and an m/z of 287.15 [M + H]^+^ (compound I in Fig. S3) was detected at day 2, suggesting a dehydrogenation of TE, while no degradation intermediates were detected in the E2 bioreactors. Similar to the small-scale enrichments, the amount of initially detectable E2 was only around 32% of the added substrate, suggesting that abiotic factors such as hormone adsorption or precipitation added to hormone removal from the medium. Unfortunately, no results are available for an uninoculated E2 control bioreactor due to a leak. Concentrations of EE2 decreased only slightly, and the decrease was not significantly different compared to an uninoculated control (Table 2; Fig. 4A). No degradation intermediates were detected in the EE2 bioreactors.

**TABLE 2:** Hormone removal rates in the initial (i.e., linear) phase as well as overall removal efficiencies in different bioreactors^*b*^

Treatment	TE		E2		EE2	
	Initial removal rate (µM * day^−1^)	Overall removal efficiency (%)	Initial removal rate (µM * day^−1^)	Overall removal efficiency (%)	Initial removal rate (µM * day^−1^)	Overall removal efficiency (%)
Uninoculated control	8.6	47.0	NA^*a*^	NA	4.9	53.0
Single hormone	54.7	100	14.5	92.0	1.9	37.0
Single hormone + lactate/acetate	11.9	100	2.6	96.0	3.0	61.0
Mixed hormones	33.5	100	22.5	100	5.0	46.5
	26.4	100	25.6	99.6	4.6	41.0
Mixed hormones + lactate/acetate	9.3	54.4	7.0	56.5	5.3	49.2
Mixed hormones + WW	23.0	100	8.9	100	2.3	27.7
	21.7	100	9.6	100	1.6	20.6
Mixed hormones + WW + lactate/acetate	26.0	100	8.7	96.5	2.3	31.3

^*a*^ NA, not available.

^*b*^ Removal efficiencies were calculated by comparing the initially measured hormone concentrations (at t_0_) to the final concentrations at *t*_end_ for each hormone and bioreactor.

The addition of 5 mM lactate and 5 mM acetate to the medium led to slower removal of TE and E2, which were completely removed only after 10–12 days (Table 2; Fig. 4B). Lactate was completely consumed within 3–4 days and acetate within 5–10 days in bioreactors with or without hormones (Fig. 4B and C), indicating that the presence of hormones did not impact microbial use of these substrates. In TE-supplemented bioreactors with lactate and acetate compound I accumulated up to day 12 of the incubation before its concentration decreased (Fig. S3A and B). In addition, two other products absorbing at 245 nm with m/z values of 285.13 [M + H]^+^ (compound II in Fig. S3) and *m*/*z* 287.16 [M + H]^+^ (compound III in Fig. S3) were detected. Comparison of retention time, absorbance, and mass spectra to authentic standards indicated that compound II was ADD and compound III androst-4-en-3,17-dion (AD), both known intermediates of TE degradation. While the ADD concentration increased up to day 14, the concentration of AD increased up to day 5 before it was depleted (Fig. S3B). Overall, the concentration of UV-absorbing compounds with absorption maxima at 245 nm decreased to around 30% after 14 days and to around 18% after 17 days of incubation (Fig. S3B), suggesting a net removal of androgenic compounds from the medium. In E2-supplemented bioreactors with lactate and acetate, two accumulating compounds with absorbance maxima at 280 nm were detected. While the mass of the first compound (IV in Fig. S3) could not be identified, compound V had an *m*/*z* of 268.88 [M-H]^-^ and comparison of its retention time, absorbance, and mass spectrum to an authentic standard indicated that compound V was estrone (E1), a central intermediate in estrogen degradation. In addition, a compound with an absorption maximum at 245 nm (VI in Fig. S3) was transiently formed, which had the same retention time, absorbance, and mass spectrum as TE. In EE2-supplemented bioreactors, minor amounts of E1 (compound V) could be detected.

**Fig 5:**
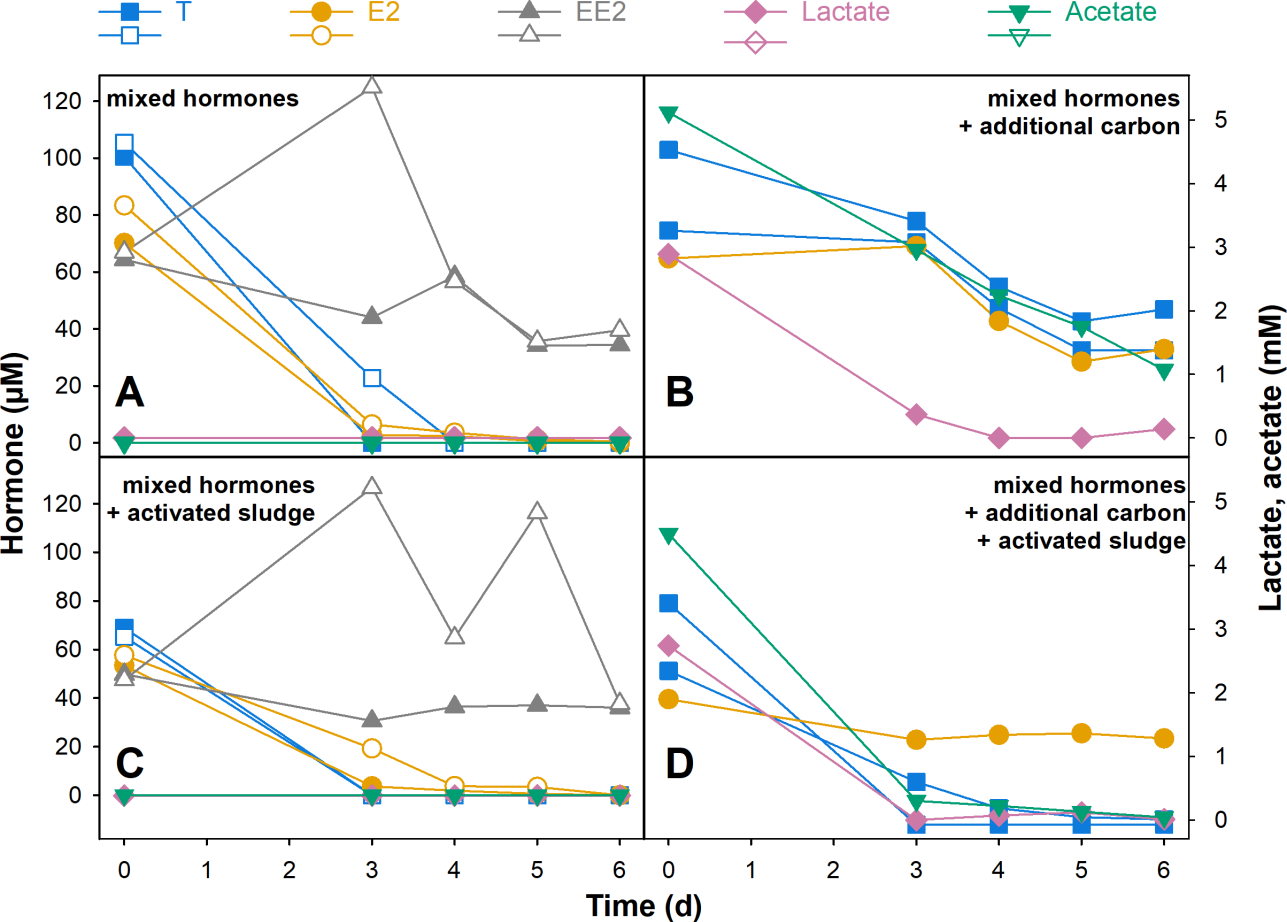
Degradation of hormones in flow-through reactors. (**A**) Duplicate reactors were supplemented with 100 µM of TE, E2, and EE2. (**B**) A single reactor was supplemented with 100 µM TE, E2, and EE2, as well as 5 mM lactate and acetate. (**C**) Duplicate reactors were supplemented with 100 µM TE, E2, and EE2, and 10% sterilized AS from a communal WWTP was added. (**D**) A single reactor was supplemented with 100 µM TE, E2, and EE2, as well as 5 mM lactate and acetate, and 10% sterilized AS was added. Open/closed symbols of the same color represent duplicate incubations.

In duplicate bioreactors that were each supplemented with a combination of all three hormones, TE and E2 were depleted within 3–4 days, while EE2 concentrations decreased only slightly (Table 2; Fig. 5A; Fig. S4B). The addition of lactate and acetate decreased hormone removal, and TE, E2, and EE2 were not depleted completely within 6 days of incubation (Table 2; Fig. 5B; Fig. S4B). Lactate and acetate consumption rates were similar to those in the single hormone bioreactors. The addition of sterilized AS from a communal WWTP did not negatively impact hormone degradation in bioreactors without lactate and acetate but increased TE and E2 hormone removal and lactate and acetate consumption rates in bioreactors with those additional carbon sources (Table 2; Fig. 5C and D; Fig. S4C and D). The androgenic degradation intermediates I and III accumulated in minor amounts during the first 6 days of incubation in bioreactors with additional carbon substrates (Fig. S4B). The estrogenic transformation product V (E1) was also detected in all bioreactors supplemented with mixed hormones (Fig. S4). While results were slightly inconsistent between the treatments, E1 concentrations seemed to reach a maximum within the first 3–4 days, after which levels either stayed constant or started to decrease (not shown), suggesting only a low net removal of estrogenic compounds from the medium.

### Microbial community composition in hormone-degrading flow-through bioreactors

**Fig 6:**
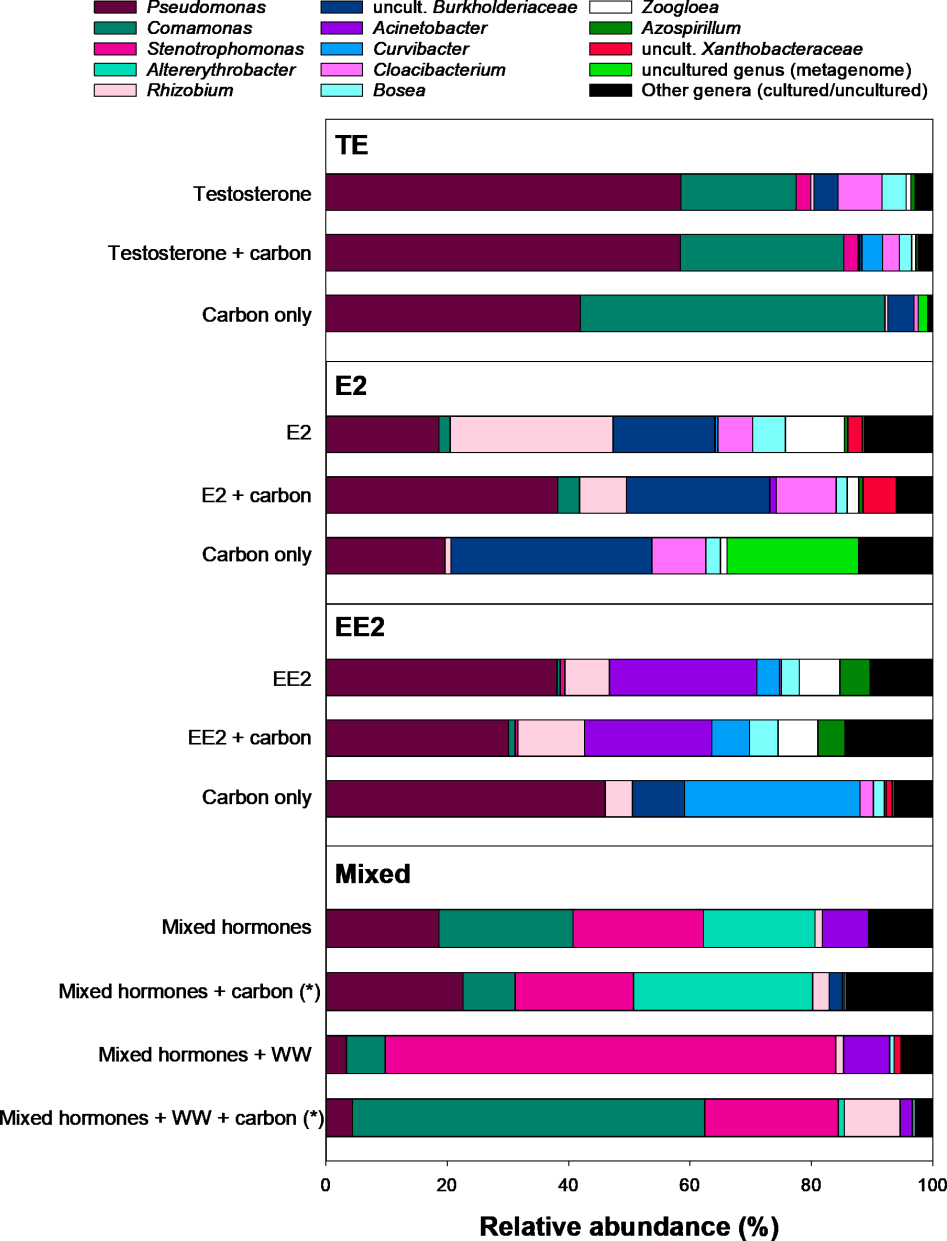
Genus-level microbial diversity in 1 L flow-through reactors and in AS from communal wastewater based on 16S rRNA gene amplicon sequencing. Genera with an average relative abundance <1.2% in the bioreactors or a relative abundance <5% in all individual bioreactor samples were grouped. Microbial communities of bioreactors with or without hormones, carbon, or AS addition are shown separately. (*) = average of two replicates. Detailed microbial community composition of AS is shown in Fig. S5.

To investigate whether the steroid hormones had an effect on the microbial community composition in the upscaled bioreactors, 16S rRNA gene amplicon sequencing with material from each bioreactor was performed. Overall, the bioreactor microbial communities were dominated by only a few bacterial genera, and the community composition and dominant genera differed between bioreactors for the different enrichment types (TE, E2, and EE2) and for the mixed hormone reactors (Fig. 6). In bioreactors inoculated with cells of a TE-enriched consortium, comprising *Comamonas* and *Pseudomonas* (TE-4 and TE-5)*,* microbial communities were dominated by members of these two genera, and community composition was similar in all three treatments (TE only, TE with additional carbon, and additional carbon without TE). In bioreactors inoculated with bioelements from E2 enrichments, microbial communities were dominated by *Pseudomonas, Rhizobium,* and a genus of the family *Burkholderiaceae. Rhizobium* had the highest abundances in the bioreactor with E2 only, while it was less abundant in the bioreactor with E2 and additional carbon, and almost absent in the bioreactor with additional carbon without E2. In the latter, an uncultured genus became abundant. In bioreactors inoculated with bioelements from EE2 enrichments, microbial communities were dominated by *Pseudomonas, Rhizobium,* and *Acinetobacter*. While *Acinetobacter* was only detected in the bioreactors in which EE2 was present, *Curvibacter* became abundant in the bioreactor with carbon only. In bioreactors supplemented with all three hormones and inoculated with bioelements from the single TE, E2, and EE2 upscale bioreactors, microbial communities were dominated by *Comamonas, Pseudomonas,* and *Stenotrophomonas,* both with and without added carbon or addition of sterilized real wastewater. In reactors without additional wastewater, members of the genus *Altererythrobacter* were also abundant, while the addition of sterilized real wastewater resulted in shifts in relative abundances, as *Stenotrophomonas* became more abundant. The AS added to the bioreactors to simulate the effect of communal wastewater on hormone removal in bioreactors hosted a very diverse microbial community (Fig. 6; Fig. S5), which was clearly different from the microbial communities that developed in the bioreactors.

## DISCUSSION

Bioaugmentation with microbes or microbial consortia that are able to break down specific micropollutants is increasingly seen as a viable option for increasing the efficiency of different water treatment systems (9, 10). In this study, biofilm-forming, hormone-degrading bacteria were enriched with low concentrations of androgen and estrogen hormones, and the hormone removal efficiency of the enrichments was tested in increasingly complex settings approaching real-life conditions of small-scale wastewater treatment systems. In the initial enrichments, removal of all four steroid hormones was reproducible and stable over several culture transfers. However, while individual androgen-degrading strains were successfully isolated from the TE and ADD enrichments, isolation of estrogen-degrading strains was not successful, as none of the obtained isolates showed consistent estrogen removal in single culture.

Most isolates from androgen enrichments belonged to the *Comamonas* genus and had closest affiliations with the species *Comamonas testosteroni*. This species contains many strains, which degrade TE and other steroids (16, 17) by a highly conserved steroid degradation pathway (7, 18). Accordingly, *C. testosteroni* has been identified before as the main androgen-degrading organism in AS (19), which corroborates the dominance of *Comamonas* isolates in the TE enrichments. Indeed, all *Comamonas* strains isolated in this study rapidly degraded TE and ADD (an intermediate in TE degradation) in single culture, as did upscaled bioreactors inoculated with *C. testosteroni* TE-4. This shows that highly efficient androgen-degrading *Comamonas* strains can be enriched from AS with low androgen concentrations in a flow-through system and be successfully reintroduced into small-scale bioreactors to degrade androgen micropollutants. In a similar study, a 3-chloroaniline-degrading *C. testosteroni* strain originating from the same ecosystem was able to establish and maintain a stable population within the native community of a semicontinuous AS system, and 3-chloroaniline was degraded in the bioaugmented setup but not without the addition of this strain (20). This confirms that bioaugmentation scenarios with members of the *Comamonas* genus can be highly effective.

The ability to produce and integrate into biofilms is considered another prerequisite to successfully use hormone-degrading microbes in real-life applications, as this limits washout of the degraders from the system and renders constant reinoculation unnecessary (21). In enrichments with androgen substrates, strong biofilm formation was repeatedly observed, and transfer of biofilm-containing carriers co-transferred androgen degradation to fresh cultures, suggesting that the androgen-degrading *Comamonas* bacteria successfully integrated into the biofilms. Indeed, *C. testosteroni* strains are known for their capability to form biofilms and to switch between planktonic and biofilm lifestyles (22). This shows that a combination of flow-through reactors along with appropriate surface materials is a suitable setup to enrich biofilm-forming, hormone-degrading microorganisms for future bioaugmentation scenarios.

While removal of E2 and EE2 was observed in the enrichments, the obtained isolates did not show consistent E2/EE2 removal in single culture, and no or only low E2/EE2 removal was observed in upscaled bioreactors. This raises the question of whether E2/EE2-degrading microorganisms were lost during the enrichment transfers, or whether significant biological degradation ever occurred in the enrichments. E2 and EE2 are highly hydrophobic compounds with a very low solubility in aqueous systems (23), which strongly adsorb to plastic materials (24). It is thus feasible that the initially observed removal of E2 and EE2 was (at least in part) caused by precipitation and adsorption rather than by biodegradation. This was also corroborated by a sterile E2 culture, which showed similar E2 removal rates as inoculated cultures. Similar findings with high adsorption rates and low biodegradation rates have been observed for E2 and EE2 in batch cultures with AS (25). However, the formation of typical estrogen degradation intermediates such as E1 in upscaled bioreactors suggests that some biological E2 and EE2 transformation activity remained in later transfer stages. Many bacteria, including members of the *Novosphingobium*, *Sphingomonas*, *Pseudomonas,* and *Rhodococcus* genera (26–30), are able to convert E2 into E1 by 17β-hydroxysteroid dehydrogenase activity. While some of these bacteria degrade E1 into less potent estrogens, others accumulate E1 as an end product. Since E1 retains significant estrogenic activity, a conversion of E2 into E1 can reduce the total estrogenic load in wastewater, but a complete elimination of E1 and E2 would be highly preferable in bioaugmented water treatment solutions.

Other isolates from estrogen enrichments were related to *Zoogloea, Pseudomonas, Pelomonas, Comamonas,* and *Methyloversatilis*. While *Zoogloeae*, *Pseudomonas,* and *Comamonas* amplicon sequence variants (ASVs) were also present in the upscaled estrogen bioreactors, *Pelomonas* and *Methyloversatilis* ASVs were absent, suggesting that they were lost in later enrichment steps. While there have been reports that some members of the *Pseudomonas* and *Comamonas* genera can degrade estrogens (31, 32) and members of the *Zoogloea* genus bile acids and androgenic steroids (33)*,* steroid degradation in *Pelomonas* and *Methyloversatilis* has not been reported. The fact that none of the isolates was able to degrade and grow with E2/EE2 in pure culture indicates that these strains (i) used other carbon sources for growth in the enrichments, (ii) co-metabolized the estrogen substrates, or (iii) associated themselves strongly with the transferred PE carrier biofilms without a major increase in their cell numbers. *Zoogloea* strains, for example, are prominent floc- and biofilm-forming microbes (34), which could explain their transfer over several enrichment passages due to strong attachment to the PE carriers. On the other hand, all isolates belong to genera that are well known for their ability to degrade complex organic substances, including aromatic and other recalcitrant compounds (22, 35–39), suggesting that they might have proliferated with organic residues that leaked from the plastic materials. Another option could be an obligate co-metabolism of the estrogen hormones mediated by cross-feeding of metabolic intermediates within the enriched community, which has been reported to occur in estrogen-spiked membrane bioreactors with active sludge (40). To study potential synergistic co-metabolism of estrogens, future enrichment studies should be accompanied by amplicon and functional metagenomics to identify obligate degradation partners, allowing for a more targeted isolation strategy of the consortia or of their individual partners. Interestingly, an androgenic (C_19_) intermediate was detected in E2 bioreactors with lactate and acetate as additional carbon sources, suggesting a methylation of E2 or E1 at C9. Such a retro-conversion of estrogens into androgens has been reported in a denitrifying *Denitratisoma* strain (41, 42), and it seems possible that a similar reaction could have been carried out by the estrogen-enriched community. This would produce intermediary androgen compounds that are easier to degrade and could be used as growth substrates by *Comamonas* and other androgen degraders.

In a control culture without estrogen hormones and in later stages of the estrogen enrichments, high planktonic growth was observed despite low E2/EE2 removal, indicating that other medium components, rather than the supplied estrogens, allowed microbial growth. Indeed, the three *Pseudomonas* isolates from the TE, ADD, and E2 enrichments showed growth in the AWW medium without using the provided steroid hormones as substrates. While some *Pseudomonas* strains are reported to degrade androgens and estrogens (31, 43, 44), steroid degradation genes are absent from most *Pseudomonas* genomes (7). Genome sequencing of the TE isolate *Pseudomonas* sp. strain TE-5 confirmed that androgen degradation genes are absent from its genome (GenBank accession GCA_033704915.1). Further studies with this strain showed that it can metabolize and grow with the AWW medium buffer Tris(2-amino-2(hydroxymethyl)propane-1,3-diol) as the only carbon and nitrogen source (45), suggesting that the isolated *Pseudomonas* strains were enriched by using Tris as a growth substrate. The ability of *Pseudomonas* to proliferate in AWW medium was also corroborated by the fact that *Pseudomonas* made up around 19%–46% of the microbial communities in upscaled estrogen bioreactors and around 42%–59% in upscaled androgen bioreactors. Organic buffers such as Tris, which can potentially serve as alternative carbon sources for microorganisms, should therefore be avoided in isolation studies and be replaced with inorganic buffers (46). These results highlight the difficulties that arise when trying to enrich microorganisms that degrade challenging micropollutants such as steroid hormones and underline the importance of choosing a suitable growth medium for bioaugmentation enrichment studies. To improve the isolation of estrogen-degrading bacteria, future enrichment strategies should consider the use of materials (boxes, tubing, carrier materials, etc.) that do not adsorb estrogenic steroid hormones, to make sure enough substrate remains in the cultures for bacterial growth. In addition, using batch cultures in glass flasks for the initial enrichment steps might increase the chance for estrogen degraders to proliferate, and an incremental increase of the estrogen concentration between enrichment transfers and longer incubation times could help to select for more robust degraders (47).

In up-scaled flow-through reactors, TE and E2 were removed within 1–2 days in the simplest setup (i.e., single hormone only) while additional carbon and/or a combination of hormones decreased removal rates. The availability of easily degradable carbon has been shown to decrease steroid degradation rates in wastewater treatment systems (25). In *Comamonas*, the expression of key TE metabolism genes was shown to be repressed in the presence of acetate and citrate (48), suggesting that catabolite repression might have been responsible for slowing down TE degradation in the upscaled bioreactors. This indicates that it might prove difficult to maintain hormone-degrading communities in bioaugmented real-life wastewater treatment applications, because wastewater typically contains high amounts of less recalcitrant carbon sources that could inhibit steroid degradation, especially since steroid hormone concentrations in communal wastewater are typically much lower than the 100 µM that were added to the bioreactors. To prioritize hormone degradation over the degradation of competing substrates, strains that do not exhibit catabolite repression should be selected for bioaugmentation. In addition, hormone-degrading strains could be paired with generalist degraders to remove competing substrates, and hormone-degrading strains could be immobilized on suitable carriers such as activated carbon to adsorb steroid hormones and to create microenvironments with higher hormone concentrations. However, while acetate and lactate decreased steroid removal rates, other organic compounds in real wastewater might provide beneficial carbon sources for microbes to foster co-metabolism of steroid substrates (49). Fittingly, sterilized real wastewater did not negatively impact hormone removal rates in mixed bioreactors, but even increased TE and E2 removal in the presence of lactate and acetate, showing that hormone removal can be improved in the presence of real AS. However, since steroid hormones sorb to organic matter (25, 50, 51), this increase might have been caused by sorption rather than degradation, and additional studies would be needed to further investigate which process dominates. Moreover, in non-sterilized wastewater strains or consortia introduced through bioaugmentation compete with the autochthonous microbiota, which is typically well adapted to the wastewater treatment systems. Many studies attempting bioaugmentation of various systems have found a failure of the introduced microorganisms to persist long term in the system (52, 53). Thus, frequent monitoring of wastewater treatment systems after bioaugmentation would be necessary, which could then identify the need to reseed the system with the desired degraders if long-term persistence cannot be established. Typical water residence times in wastewater treatment systems are often less than 24 h; that is, the removal rates observed in the bioreactors would not be sufficient to completely remove the added steroid hormones in a real-life scenario. To avoid this problem, real-life bioaugmentation scenarios for steroid hormones should therefore select microbes with the highest degradation rates and the lowest washout rates to increase biological removal efficiencies. In addition, bioreactors that allow high biomass loads, such as membrane bioreactors or moving bed biofilm reactors, should be used, and hydraulic retention times need to be optimized for individual target compounds. Other treatment techniques, such as activated carbon or advanced oxidation processes, can be combined with targeted bioaugmentation to further increase pollutant retention times and to support biodegradation. In this study, 100 µM hormone concentrations were used, which is still at least 100 times more than typical hormone concentrations in wastewater (54).

The amplicon sequencing results indicated that other microbes than the isolated strains were also present in the upscaled bioreactors. Some of these taxa contain members that degrade steroids, such as the *Burkholderiaceae* family (7), and the *Acinetobacter* and *Altererythrobacter* genera (55–58), suggesting that they might have been involved in steroid removal in the bioreactors, but did not yield any isolates on solid media. These findings offer the potential for a more targeted strategy to specifically enrich and isolate these genera for added steroid hormone degradation capacities. In future studies, functional gene or metagenome analysis focusing on known steroid degradation genes should be considered to get deeper insight into the steroid degradation potential of enriched communities. Alternatively, some of the taxa might have used other carbon sources and/or strong attachment to biofilm carriers to persist in the microbial community throughout the enrichment and bioreactor transfers. Similar to *Pseudomonas*, some *Rhizobium* strains encode genes for Tris degradation (45), indicating that the *Rhizobium* ASVs might have persisted using Tris as a growth substrate. *Stenotrophomonas* bacteria are known for their ability to form biofilms and to degrade xenobiotic compounds (59, 60).

### Conclusions

In summary, the results of this study show that microbes can help to remove low concentrations of steroid hormones from wastewater, confirming that bioengineering of water treatment systems with steroid-degrading microbes is a promising technique to increase the removal of these micropollutants. This study also highlights potential pitfalls of isolating microorganisms for bioaugmentation purposes and shows that additional research and careful planning are needed for the implementation of bioengineered systems for the removal of micropollutants.

## MATERIALS AND METHODS

### Enrichment of steroid hormone-degrading microorganisms in flow-through reactors

**Fig 7:**
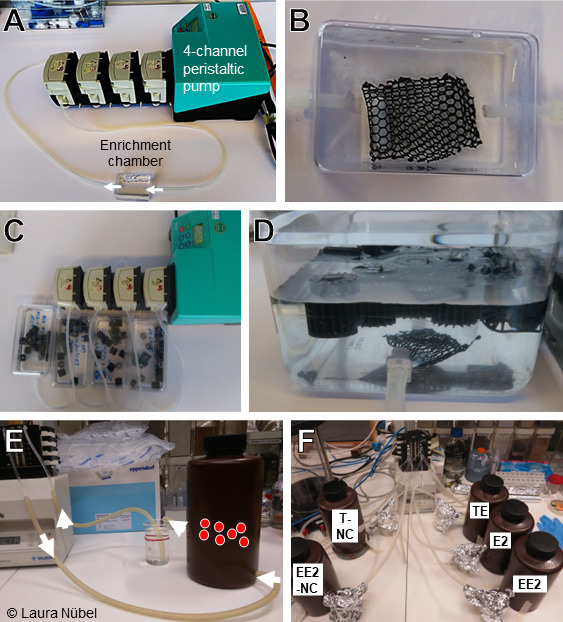
Flow-through systems for enrichment and upscaling. (**A**) Initial enrichment stage: 35 mL enrichment chambers connected to a peristaltic pump, flow-through 50 mL/min; (**B**) close-up of an enrichment chamber: PE-plates for biofilm growth were located inside a PE net to prevent washout of PE-plates; (**C**) upscaling to 250 mL chambers and transfer to PP-bioelements; (**D**) close-up; (**E**) example of 1 L system: PP-bioelements in flow-through reactor, medium circulated at 25 mL/min through reactor and overflow container, overflow container served to improve aeration and allow for sampling of medium during the experiment; (**F**) 1 L flow-through reactors in initial round of upscaling: three reactors supplemented with individual hormones (TE = testosterone, E2 = estradiol, EE2 = ethinyl estradiol), two reactors with sterile controls (T-NC = testosterone negative control, EE2-NC = ethinyl estradiol negative control). During operation, enrichment chambers and reactors were covered to avoid exposure to light.

Hormone-degrading microorganisms were enriched from AS, which was sampled from the AS basin at the WWTP Münster-Coerde on 21 June 2019. An AWW medium was used for all cultures. AWW contained 5 mM Tris, 3.57 mM NH_4_Cl, 1.07 mM urea, 0.74 mM NaNO_3_, 0.12 mM NaCl, 0.036 mM CaCl_2_ × 2H_2_O, 0.017 mM MgSO_4_ × 7H_2_O, 0.0098 mM K_2_HPO_4_ × 3H_2_O, 0.0042 mM NaH_2_PO_4_ × H_2_O, and trace elements and vitamins (61) and was adjusted to a pH of 7 (Table S1). Steroid-degrading microorganisms were enriched in individual flow-through reactors (Fig. 7) consisting of silicone tubing and polystyrene enrichment chambers (56 × 36 × 26 mm) each containing 35 mL of AWW medium (Fig. 7A). The enrichment chambers were connected to a peristaltic pump with four pump heads (Watson-Marlow GmbH, Rommerskirchen, Germany) and medium was pumped at a rate of 50 mL/min to select for biofilm-forming microorganisms. Ten PE plates (4.0 × 4.0 × 0.125 mm) were added into each chamber to provide a colonization surface. The PE carrier plates were enclosed by a PE net and weighted down by a magnetic stir bar to avoid their washout of the system (Fig. 7B). All components were sterilized by a 10 min incubation step in 70% (wt/wt) ethanol followed by a 10 min UV treatment (Philips TUV 30W T8) in a laminar flow hood. The silicone tubing was sterilized by autoclaving prior to assembly. Enrichment media were supplemented with a single hormone (i.e., TE [Acros Organics], ADD, E2 [Sigma-Aldrich], EE2 [Acros Organics]) as carbon source at a concentration of 100 µM and incubated at room temperature. ADD was produced from lithocholic acid by incubating *Pseudomonas stutzeri* Chol1 under anoxic conditions with nitrate as an electron acceptor according to Philipp et al. (62) and extracting ADD from the culture medium with dichloromethane. Purified ADD was resuspended in isopropanol, and ADD purity was assessed with high-performance liquid chromatography-mass spectrometry (HPLC-MS) analysis (not shown). Growth of planktonic cells was monitored by determining the optical density at 600 nm (OD_600_). Growth of biofilm formation was monitored by macroscopic and microscopic observations. Steroid hormone removal was monitored by organic extraction of the culture supernatants and HPLC analysis.

Enrichments were inoculated with 1 mL AS, and enrichments were transferred into fresh medium once the initially supplied hormones were depleted. For this, five PE carrier plates were rinsed in sterile deionized water and transferred into fresh medium supplied with the same hormone as previously. Androgen enrichments were transferred four times, and estrogen enrichments were transferred fifteen times. In addition, a single uninoculated control was set up with 100 µM of E2 to test for abiotic E2 removal, and one control culture without hormone substrate was inoculated from the fourteenth transfer of the E2 enrichment.

### Isolation of hormone degraders from flow-through enrichments

After three transfers, biofilm-forming hormone degraders were isolated on agar plates containing the same hormone (Fig. 1). For this, one PE element displaying pronounced biofilm formation was removed from each flow-through reactor, transferred to 1 mL AWW medium, and vortexed briefly to suspend cells from the biofilm. 100 µL of the resulting suspension was transferred to AWW agar plates containing 100 µM of the target hormone. Plates were incubated at room temperature in the dark, and colonies were selected and transferred to fresh plates at least three times to obtain pure cultures.

### Characterization of hormone-degrading microorganisms

The hormone degradation potential of the isolated microorganisms was tested in glass tubes in 5 mL AWW medium containing 500 µM of the respective isolation hormone in the dark at room temperature and 200 rpm, and cultures were sampled every 1–3 days to determine OD_600_. Hormone concentrations in the culture supernatants were determined at the beginning and the end of each experiment.

Isolates were identified by 16S rRNA gene sequencing. Genomic DNA of selected isolates was extracted using the Puregene Yeast/Bacteria kit B (Qiagen, Hilden, Germany) according to the manufacturer’s instructions for Gram-negative bacteria. 16S rRNA genes were amplified using the common 27F/1492R primer pair, purified using the GeneJET PCR Purification Kit (Thermo Fisher Scientific), and sequenced from both ends at Microsynth AG (Balgach, Switzerland).

The obtained sequences were analyzed using the SnapGene Viewer (https://www.snapgene.com/snapgene-viewer/). Sequence quality was checked, and only parts of the sequences with strong, unambiguous base calls were used in downstream analyses. Consensus sequences of forward and reverse reads were generated using Serial Cloner (http://serialbasics.free.fr/Serial_Cloner.html). Closest related sequences were determined using BLASTn against the 16S ribosomal RNA sequence database of NCBI, and lowest common ancestors were determined using the SINA search and classification algorithm at SILVA using standard settings. Sequence alignments were created from the isolates and their closest related sequences using the Muscle algorithm in MEGA (version 11.0.13) (63), and maximum-likelihood phylogenetic trees were calculated in MEGA.

### Upscaling of hormone-degrading enrichments

For the initial upscaling, three 1 L bioreactors were set up containing 100 µM of either TE, E2, or EE2. The 1 L reactors were constructed from opaque 2 L polypropylene (PP) bottles and contained 60 PP bioelements (RK BioElements, Skive, Denmark; Fig. 7E and F) as biofilm growth surfaces. Bottles and all other equipment were sterilized by autoclaving prior to setup. AWW medium was pumped into the reactors near the bottom at a flow of 25 mL/min and left the reactors through an overflow pipe into a small glass reservoir. From this reservoir, the medium was returned to the reactor. The reservoir served to ensure better aeration of the medium and to allow sampling at regular intervals to determine OD_600_ and hormone concentrations. Dissolved oxygen was measured occasionally using a handheld device (WTW FDO 925) and ranged from 5.8 to 7.2 mg/mL.

The TE-containing bioreactor was inoculated with a consortium of TE isolates, which was originally classified as a pure culture of a *Zoogloea* sp. strain that showed TE degradation (not shown) and was therefore selected to inoculate the upscaled TE bioreactor. However, it was subsequently revealed that this isolate was a consortium comprised mainly of a TE-degrading *C. testosteroni* strain (TE-4; Table 1; Fig. S2) and a *Pseudomonas* sp. strain (TE-5; Table 1). The latter grew in close association with strain TE-4 but did not grow with TE alone (Fig. S2) (45). Since none of the isolated strains from the small-scale E2 and EE2 enrichments showed consistent hormone degradation in liquid culture (Fig. S2), an intermediary enrichment step was set up for these cultures in medium-scale bioreactors containing 250 mL AWW medium (Fig. 1) as described above for the small-scale enrichment cultures, except that the PE net with the PE carrier plates was replaced by 16 PP bioelements per reactor (RK BioElements, Skive, Denmark; Fig. 7C andD). The 250 mL bioreactors were inoculated with one PE net with biofilm-containing PE plates from the eighth small-scale enrichment transfer containing the same hormone substrate. Once 250 mL cultures showed an increase in turbidity and biofilm growth, five biofilm-containing bioelements were used to inoculate the respective 1 L reactors containing E2 and EE2 (Fig. 1). Single hormone bioreactors were run for 10 days and sampled regularly (every 1–3 days). In addition, uninoculated controls were set up with 100 µM of TE, E2, or EE2 but with sterile bioelements to test for abiotic steroid hormone removal (Fig. 7F). Unfortunately, due to a leakage of the E2 bioreactor, only results for TE and EE2 are available.

To better simulate the conditions in real-life wastewater treatment applications, two further large-scale incubations with modified substrate combinations were set up as described above. To test the effect of alternative carbon sources commonly found in wastewater on hormone removal, three 1 L cultures were set up containing 100 µM of TE, E2, or EE2, and additionally 5 mM lactate and 5 mM acetate. Three 1 L bioreactors containing only lactate and acetate, but no steroid hormones, served as controls. These bioreactors were inoculated with five PP bioelements collected on day 10 from the initial 1 L reactors containing the respective hormone and were incubated for 17 days. Additionally, six 1 L bioreactors were set up, all of which contained a combination of 100 µM of all three steroid hormones. Three bioreactors were supplemented with 1/10 of their volume with autoclaved AS (120°C, 60 min) collected from the membrane bioreactor of a municipal WWTP (collected on October 31, 2019, Taskila Jätevedenpuhdistamo, Oulu, Finland), the other three bioreactors did not contain AS (Fig. 1). One bioreactor of each set was supplemented with 5 mM acetate and 5 mM lactate. All six bioreactors were inoculated with one PP bioelement collected on day 10 from the initial scale-up bioreactors (containing TE, E2, or EE2) and were incubated for 6 days. Samples from all upscale cultures were taken regularly and steroid hormone removal was monitored by organic extraction of the culture supernatants and HPLC-MS analysis.

### 16S rRNA gene amplicon sequencing of bioreactor microbial communities

The microbial community composition in the upscaled 1 L bioreactors (15 samples) as well as in the wastewater from Taskila WWTP (one sample) was assessed through 16S rRNA gene amplicon sequencing. 2–3 mL of medium was taken from the bioreactors at the end of the respective incubation time. Cells in the medium were pelleted by centrifugation, the supernatant was discarded, and the pellet was resuspended in 200 µL sterile dH_2_O. DNA was extracted using the Bacterial Genomic DNA Isolation Kit (Norgen Biotek) according to the manufacturer’s instructions. The V4 region of prokaryotic 16S rRNA genes was amplified using the universal primers 515F (GTGYCAGCMGCCGCGGTAA) (64) and 806R (GGACTACNVGGGTWTCTAAT) (65). After a primary PCR using primers 515F/806R, barcodes and sequencing adaptors were attached to the amplicons in a secondary PCR with 10 cycles. Primers in the secondary PCR were M13-515F: IonA-M13 (in 1:10 ratio) and 806 R-P1. PCR products were purified using the AMPure XP purification system (Beckman Coulter Life Sciences) and pooled in equimolar concentrations. Amplicons were sequenced on the IonTorrent PGM as previously described (66).

Obtained sequences were analyzed in qiime2 (67). Raw sequence data were imported (qiime tools import) and demultiplexed (cutadapt [68]). ASVs were obtained by denoising imported sequences using dada2 (69) using the following parameters: 38 bp trimming at 5′ end to remove primers, trimming at 3′ end to a remaining length of 250 bp, chimera detection method “consensus.” Taxonomy was assigned to ASV using the Silva 132 classifier. Relative taxa abundance was calculated on the genus level.

### Analytical techniques

Steroid hormones were extracted from culture samples using the organic solvent dichloromethane. 500 µL culture samples was added to an equal volume of dichloromethane and shaken vigorously for 5 minutes. Phases were allowed to separate, and the organic phase was transferred to a fresh tube. The extraction was repeated with fresh dichloromethane, and the organic extracts were combined. Dichloromethane was evaporated under a gentle nitrogen stream, and steroid hormones were resuspended in 500 µL acetonitrile for subsequent HPLC or HPLC-MS measurements. Hormone quantification in flow-through enrichments and in single-strain cultures was performed using an Agilent 1200 HPLC system equipped with a UV-visible light diode array detector. Hormone substrates and potential degradation intermediates were identified and quantified at their respective absorption maxima of 245 nm for TE and ADD and other products with Δ^1^- or Δ^1/4^-3-keto structures of the A-ring and at 280 nm for E2 and EE2 and other products with aromatic A-rings. Quantification and mass spectrometric analysis of hormones from larger-scale bioreactors were performed on a Dionex Ultimate 3000 HPLC system equipped with an UV-visible light diode array detector (detection at 245 and 280 nm) and an AmaZon speed ion trap mass spectrometer (Bruker). Both systems were equipped with Eurospher II 100-5 C18 reverse phase columns (150 × 3 mm; Knauer, column temperature set to 25°C). Ammonium acetate buffer (10 mM) containing 0.1% formic acid (pH 3.2, eluent A) and acetonitrile (eluent B) was used as eluents with a flow rate of 0.3 mL/min, applying a gradient starting with 10% eluent B for 2 min, increasing to 90% eluent B within 22 min, remaining at 90% eluent B for 1 min, and returning to 10% eluent B within 1 min, followed by an equilibration of 5 min. For mass spectrometry, samples were analyzed with electron spray ionization in alternating ionization mode with a capillary voltage of 4,000 V, a plate offset of 500 V, a nebulizer pressure of 22.5 psi, a dry gas flow of 12 L/min, and a dry gas temperature of 200°C. ADD, TE, E2, and EE2 standards with concentrations between 10 µM and 100 µM were used for calibration.

Organic acids were quantified from 0.2 µm filtered water samples taken from the reactors using an Agilent 1200 HPLC system equipped with an Aminex HPX-87H column (300 × 7.8 mm; Biorad) and a UV-visible light diode array detector (detection at 210 nm) using a mobile phase of 4 mM H_3_PO_4_, a column temperature of 60°C and a flow rate of 0.6 mL/min. Organic acid standards with known concentrations were used for calibration, and selected samples were spiked with known concentrations (10–5,000 µM) of acetate or lactate to assess potential peak drift and recovery.

## Data Availability

Raw amplicon sequence reads have been deposited in the European Nucleotide Archive (ENA) under accession number PRJEB86513.

## SUPPLEMENTAL MATERIAL

Supplemental figures spectrum.00649-25-s0001.pdf Fig. S1 to S5.

Table S1 spectrum.00649-25-s0002.xlsx Composition of the AWW used in flow-through bioreactors.

ASM does not own the copyrights to Supplemental Material that may be linked to, or accessed through, an article. The authors have granted ASM a non-exclusive, world-wide license to publish the Supplemental Material files. Please contact the corresponding author directly for reuse.
