# Supplementary material for: Enrichment and characterization of steroid-degrading microbes for targeted removal of steroid hormone micropollutants in small-scale wastewater treatment solutions
Source: Microbiol Spectr. 2025 Sep 25;13(11):e00649-25. doi: 10.1128/spectrum.00649-25 (PMC12584686; doi:10.1128/spectrum.00649-25)
Supplement: Supplemental figures — Fig. S1 to S5. [file spectrum.00649-25-s0001.pdf]

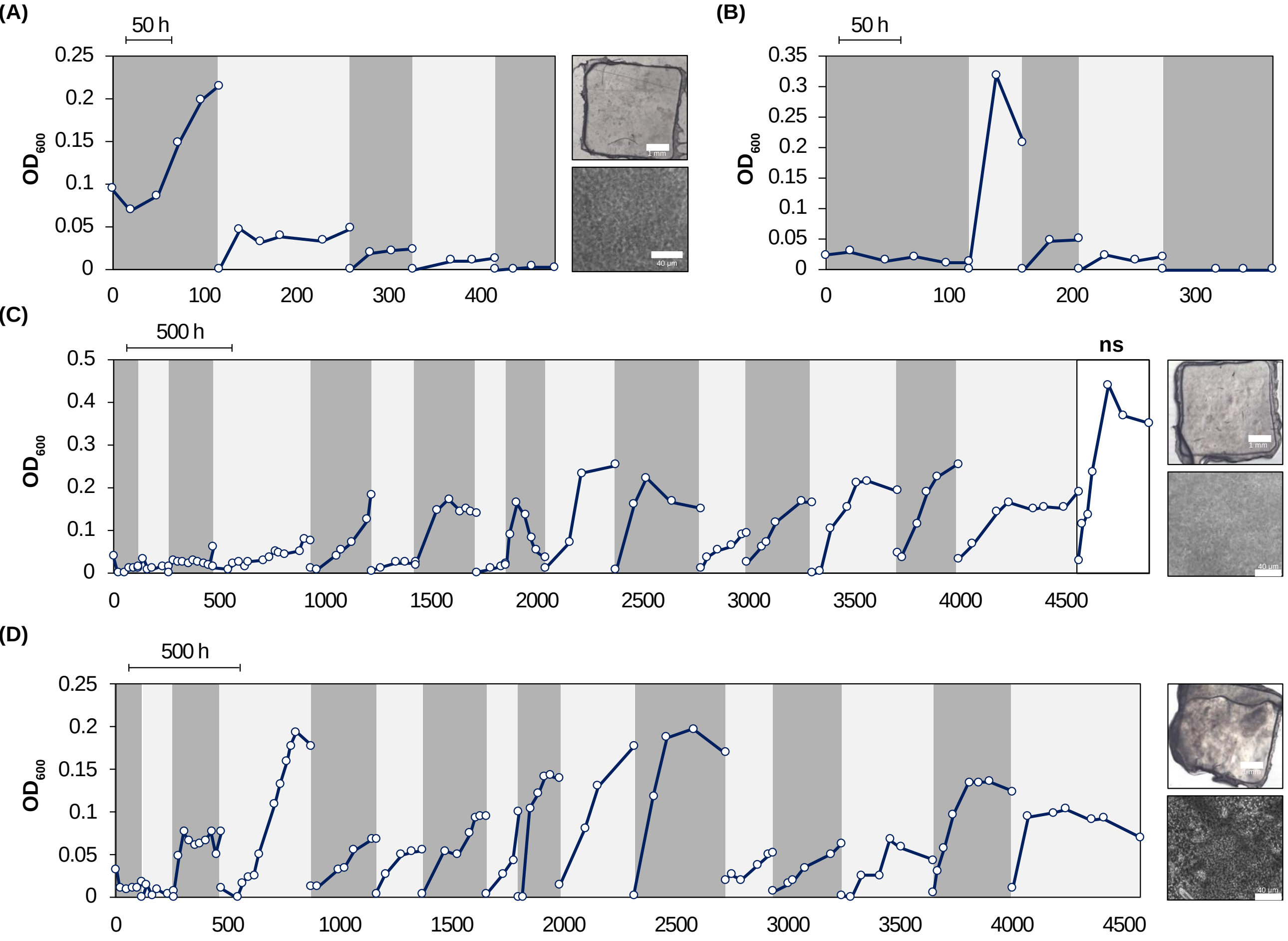

Figure S1: Growth of enrichment cultures in flow-through bioreactors with (**A**) TE, (**B**) ADD, (**C**) E2, and (**D**) EE2 in flow-through reactors for up to 4 transfers for androgen substrates and up to 15 transfers for estrogen substrates. Consecutive transfers are marked alternately in grey background color. For the E2 enrichment, a control without substrate (ns) was setup in parallel with the 14th transfer and is marked with a white background. Planktonic growth was measured as OD_{600} and biofilm growth is exemplified by macro- and microscopic pictures of selected PE carrier plates.

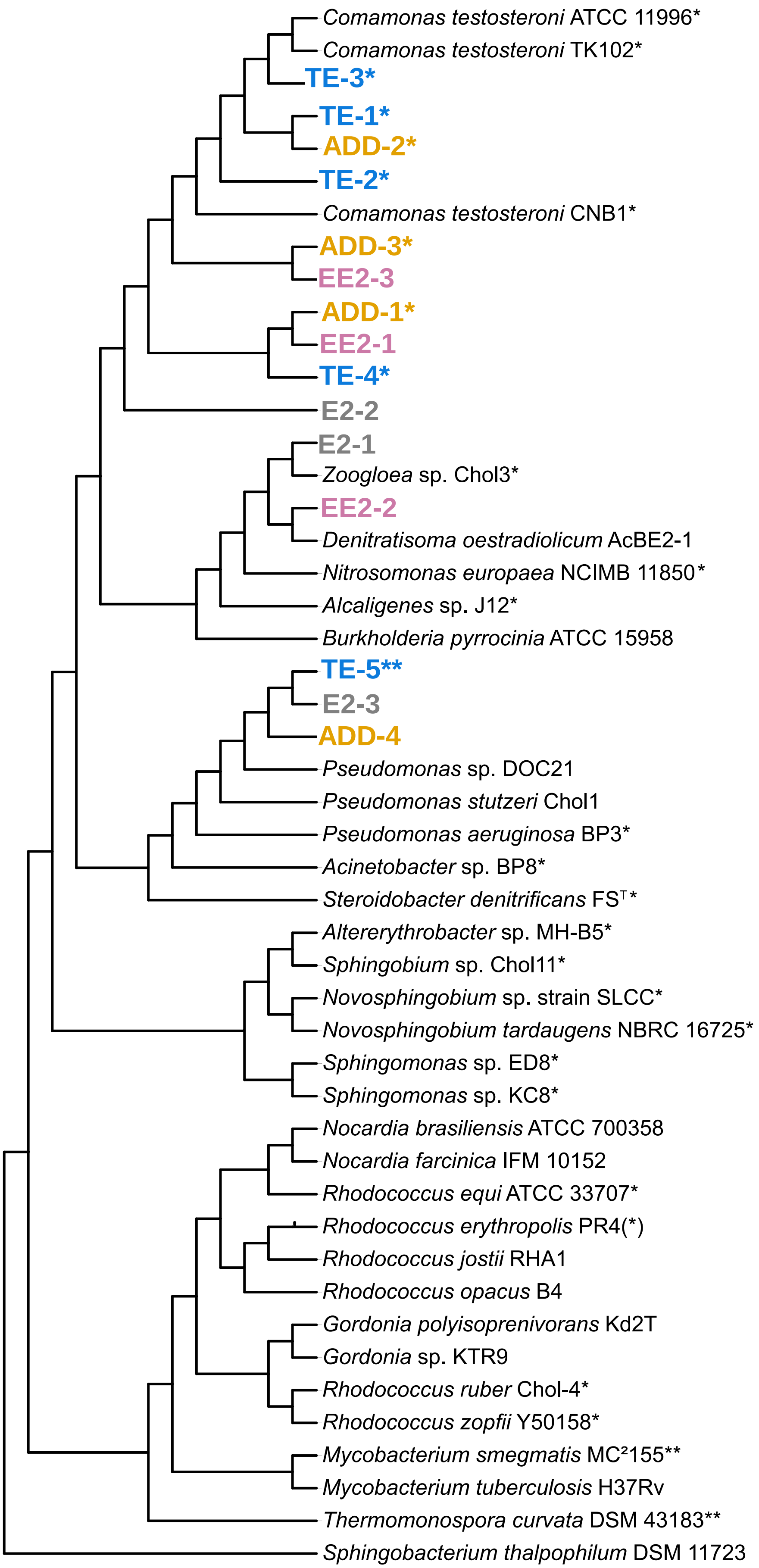

Figure S2: Maximum-likelihood tree of 16S rRNA genes of strains isolated with TE (blue), ADD (orange), E2 (gray), and EE2 (pink) and of known steroid-degrading strains. Strains that have been described to degrade steroid hormones are marked with *, to partially degrade hormones with (*), and to transform hormones with **. The tree was created in MEGA 11.0.13 with *Sphingobacterium thalpophilum* as the outgroup.

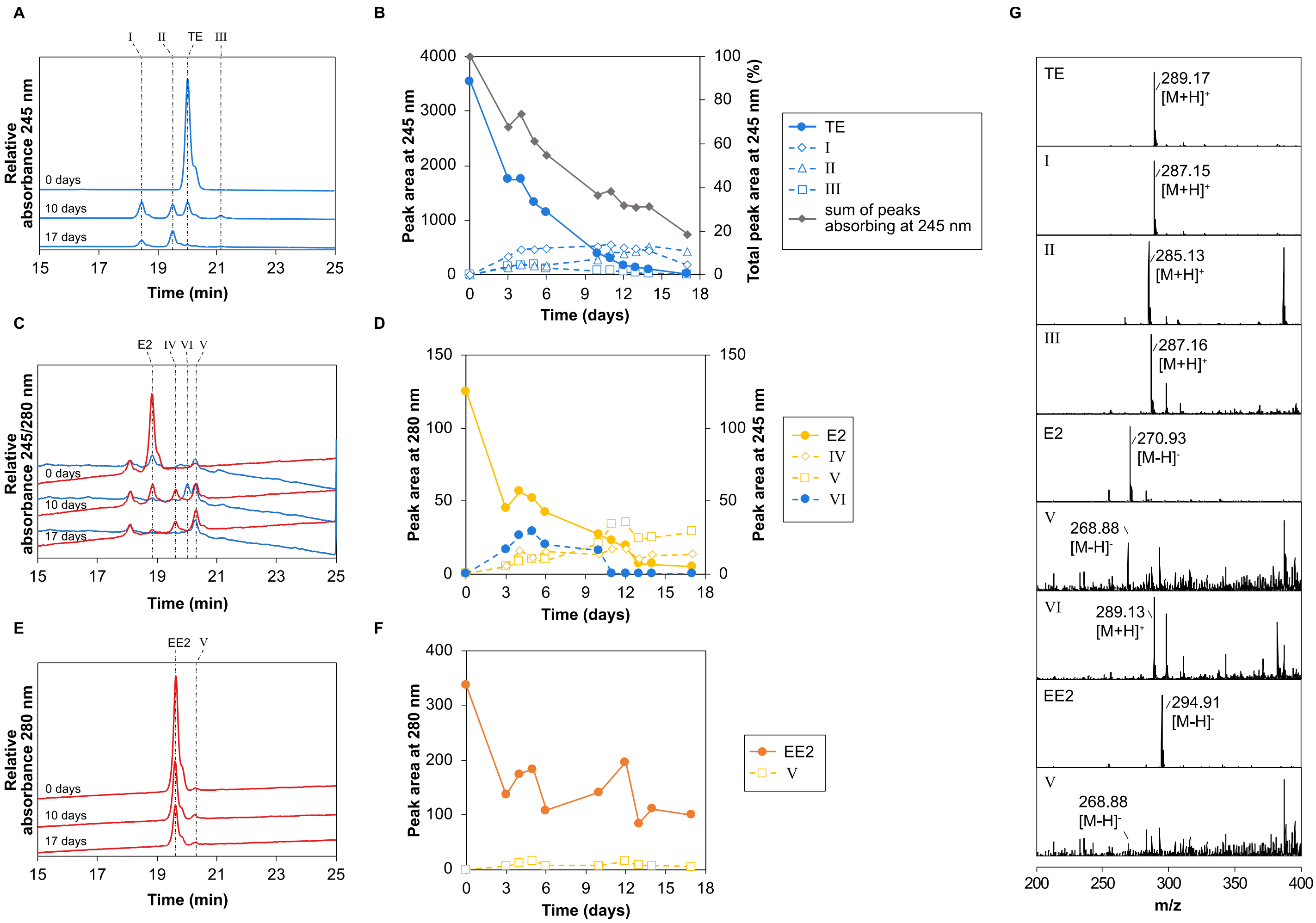

Figure S3: Identification and quantification of transformation intermediates of TE, E2, and EE2 in up-scaled bioreactors. **(A)** Representative UV-chromatograms at 245 nm of samples from a TE supplemented bioreactor spiked with lactate and acetate. Identified TE transformation products I, II, and III with 245 nm absorption maxima are marked with dashed lines. **(B)** Quantification of TE and compounds I, II, and III at 245 nm and of the sum of their peak areas (grey diamonds). **(C)** Representative UV-chromatograms of samples from an E2 supplemented bioreactor spiked with lactate and acetate at 245 nm (blue lines) and 280 nm (red lines). Identified E2 transformation products IV and V with 280 nm absorption maxima and product VI with a 245 nm absorption maximum are marked with dashed lines. **(D)** Quantification of E2 and compounds IV and V at 280 nm and of compound VI at 245 nm. **(E)** Representative UV-chromatograms of samples from an EE2 supplemented bioreactor spiked with lactate and acetate at 280 nm. Product V is marked with a dashed line. **(F)** Quantification of EE2 and compound V at 280 nm. **(G)** Representative mass spectra of TE, E2, and EE2 substrates and of compounds I – VI. TE and TE-like compounds were analyzed in positive ionization mode and molecular ion peaks are labelled. Aromatic compounds were analyzed in negative ionization mode and molecular ion peaks are labelled.

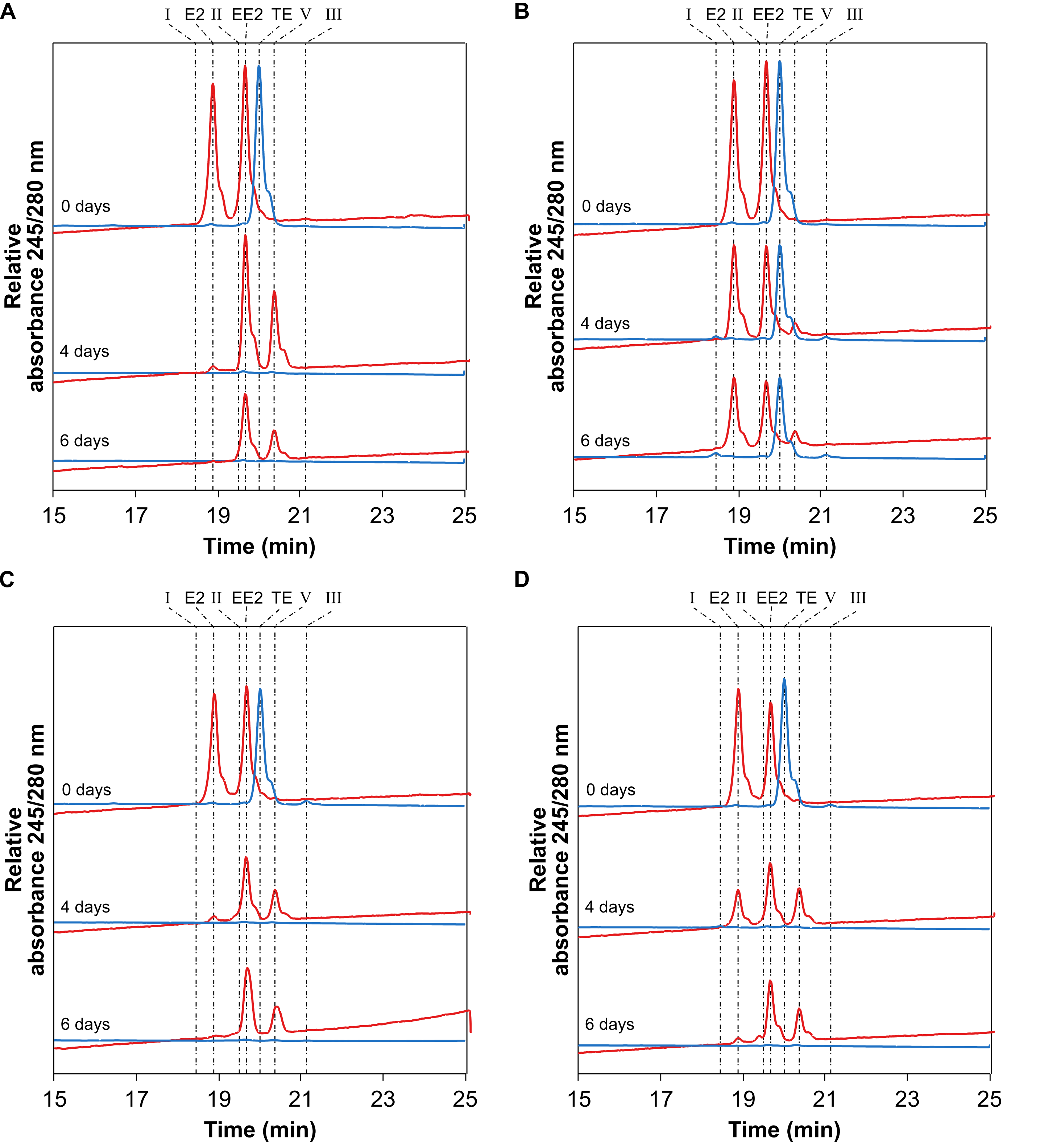

Figure S4: Identification of hormone transformation intermediates of TE, E2, and EE2 in up-scaled bioreactors supplemented with a combination of all three hormones. Representative UV-chromatograms of samples from **(A)** a bioreactor without additional carbon or wastewater, **(B)** a bioreactor supplemented with lactate and acetate without additional wastewater, **(C)** a bioreactor without additional carbon supplemented with real wastewater, and **(D)** a bioreactor supplemented with lactate and acetate and with real wastewater at 245 nm (blue lines) and 280 nm (red lines). Hormone substrates and putative transformation products are marked with dashed lines and roman numerals correspond to compounds identified in Fig. S3.

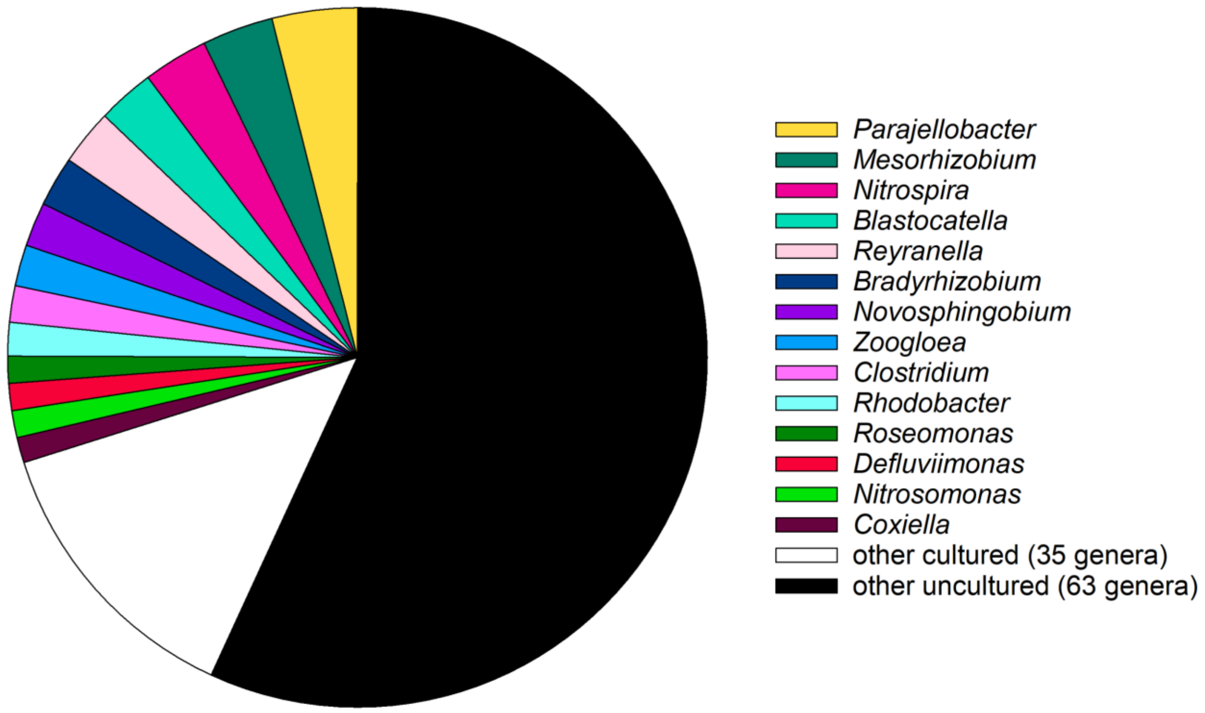

Figure S5: Genus-level microbial community composition in activated sludge from a communal wastewater treatment plant. Genera with a relative abundance < 1% were grouped.
